# COVID-19: Physical Activity Behavior Change among Athletes in Québec (Canada)

**DOI:** 10.3390/ijerph192113853

**Published:** 2022-10-25

**Authors:** Pascale Marceau, Frank Pons

**Affiliations:** Faculty of Business Administration, Laval University, 2325 Rue de la Terrasse, Québec, QC G1V 0A6, Canada

**Keywords:** COVID-19, pandemic, confinement, physical activity, well-being, athletes, Quebec

## Abstract

The context of the COVID-19 pandemic imposed unprecedented restrictions. Within Canada, which is among the most stringent countries in terms of sanitary rules, Québec was among the provinces that imposed the strictest sanitary measures. The impacts of some measures were felt the most among athletes since they made it difficult, if not impossible, to practice their sports. This article therefore aimed to (1) evaluate the impact of the pandemic on the athletes’ overall level of physical activity, (2) look at the relationship between overall level of physical activity and the level of psychological well-being and (3) analyze post-pandemic physical activity intentions. For this purpose, an online survey was conducted among 1456 athletes aged 3 to 61 years old. The results of this study show that the limitations imposed during the pandemic led to half of athletes decreasing their overall level of physical activity, leading to a deterioration in their psychological well-being (F(2.1438) = 54.707, *p* < 0.001). The current research provided further evidence that it is essential to implement strategies that favor practicing physical activities in a pandemic context. Furthermore, since almost all individuals who increased their practice of wheeled sports during the pandemic intend to continue after the pandemic, this is a great opportunity to promote active transportation among athletes by ensuring that the perception of the benefits associated with it does not decrease with time.

## 1. Introduction

At the beginning of 2020, a new coronavirus (COVID-19) was identified in Wuhan, China [1]. On 11 March 2020, the World Health Organization (WHO) officially declared that this COVID-19 pandemic was considered to be a global pandemic [1]. Following this declaration, numerous sanitary measures were implemented throughout the world. These measures, which included border closings, confinement, social distancing and the closing of non-essential businesses had a strong social impact. According to published statistics, Canada is among the most stringent countries in terms of sanitary rules [2]. Within Canada, Québec was among the provinces that imposed the strictest sanitary measures [2,3]. These measures included a curfew on citizens and the implementation of a vaccination passport.

Despite the fact that the different sanitary measures implemented ultimately aimed to slow the spread of the virus and protect the health of the population, the fact remains that they also led to undesirable effects, particularly in terms of practicing sports and physical activities. In fact, following the health emergency declared on 13 March 2020 in Québec, several public health measures were adopted and, as presented in Appendix A, a number of them had a direct impact on the possibilities for Quebecers to engage in physical activities [4]. In summary, over a period of about 19 months, between the beginning of the health emergency (13 March 2020) and the end of the present data collecting (13 October 2021), the practicing of most team sports was interrupted or regulated for a total period of over eleven months (from 15 March 2020 to 8 June 2020 during the first wave and from 8 October 2020 to 11 June 2021 during the second and third wave). Practicing individual sports was interrupted or regulated for a total period of more than seven months (from 15 March 2020 to 20 May 2020 during the first wave and from 8 October 2020 to 26 March 2021 during the second and third wave). In addition to the measures directly concerning the practicing of virtually all sports activities, other measures may have increased the sedentary behavior of individuals by forcing them to work or take courses remotely and by encouraging them to restrict their movements, for example.

Individuals who practiced the now regulated sports and physical activities inevitably had to adapt their training in order to maintain the same level of activity while respecting the various measures implemented. Athletes were therefore more directly affected by the measures that interrupted or regulated team or individual sports. It is even more important to study the impact of the pandemic on the level of physical activity knowing that before the pandemic and its restrictions, more than one-quarter of the global adult population (1.4 billion people) were not active enough [5].

The primary objective of this study is to better understand the changes in behavior in terms of athletes’ physical activities in the context of the global pandemic. More specifically, this study aims to measure the perception that athletes have as to the impact of the pandemic on their overall level of physical activity. In other words, do they estimate that their overall level of physical activity remained the same, decreased or increased? Furthermore, since physical activity can greatly contribute to an individual’s feeling of well-being [6], this article looks at the relationship between athletes’ overall level of physical activity and their level of psychological well-being. More specifically, it is expected that a reduction in the level of overall physical activity has a negative impact on athletes’ well-being. The next aim is to concretely identify the changes that took place in the practice of physical activities. Finally, this study aims to evaluate if the changes in practicing physical activities resulting from the pandemic are more temporary or permanent. In other words, do athletes intend to maintain their new behavior in terms of practicing physical activities after the pandemic?

## 2. Materials and Methods

### 2.1. Design

This study was conducted through a self-administered online questionnaire on the *Limesurvey* platform. The questionnaire was distributed through the email address base held by a sports federation overseeing the practice of soccer in Québec. The data collection was part of a larger research project. The data collection tool twinned the questions from another study with those of the current study, for a total of 82 questions, of which 37 questions directly concerned this study.

As a pretest, 1500 invitations were sent on 3 September 2021. Next, after having validated the psychometric qualities of the measuring tool without having made any modifications, 12,500 other invitations were sent on 1 October 2021. This date was chosen since it coincided with the gradual return to sports activities so that the respondents’ intentions were likely to better reflect reality.

### 2.2. Sampling

With a goal of better understanding the behavioral changes in terms of athletes’ physical activities in the context of a global pandemic, the study population is composed of soccer players. This choice is justified by three main reasons. As part of the team sport category, soccer was disrupted by the sanitary measures for the longest period. In addition, soccer players have a diversified sociodemographic profile, whether it be in terms of gender, age, income, level of education, etc. Finally, soccer is the most popular and most practiced sport in the world, which improves the external validity of the study. In the context of this study, the term “athlete” encompasses all soccer players of different levels, whether they play soccer for fun or for competition. Questions about the level of competition of the athlete were nevertheless asked in order to identify potential significant differences between these different categories of athletes.

### 2.3. Measures

#### 2.3.1. Physical Activity

The list of questioned activities is drawn from the Québec Survey on Physical Activity and Sports, 2018–2019 [7], which is an adaptation of a questionnaire used in various other studies e.g., [8,9]. Among the forty activities measured in this survey, only those that were relevant in a pandemic situation and the restrictions that accompany them were retained for the purposes of the present study. The selected activities belong to the leisure physical activity or transportation physical activity [10] categories. Hence, the questions concerning physical activities focus on the following six activities, for pleasure or to move: (1) walking; (2) wheeled sports, such as bicycling, skateboarding and scootering, etc.; (3) outdoor activities, such as skiing, hiking, etc.; (4) home exercise with or without equipment; (5) jogging; and (6) swimming.

The level of physical activity was measured with the help of subjective measures concerning, in a first step, the respondents’ perceptions about their level of physical activity during the pandemic compared to before the pandemic, considering that the pandemic started during the health emergency declared on 13 March 2020. In a second step, it involved measuring the respondents’ intentions with respect to these activities when all health restrictions would be lifted.

#### 2.3.2. Well-Being

The level of psychological well-being was evaluated using a French adaptation of the World Health Organization Well-Being Index (WHO-5) [11]. Each of the five items was evaluated on a scale from 5 (always) to 0 (never), with a theoretical maximum score of 25. Following the recommendation of Topp, Otergaard, Sondergaard and Bech [11], the total score was multiplied by four, since this type of measure is conventionally expressed as a percentage. An overall score from 0 to 100 in WHO-5 was thus used during the data analysis. The psychometric analysis of the scale demonstrated a good internal coherence (α = 0.869) [12].

## 3. Results

### 3.1. General Characteristics of the Sample

All of the statistical analyses were performed using the IBM SPSS Statistics 21 software. A total of 1750 respondents completed the questionnaire between 3 September and 13 October 2021. By subtracting the 294 respondents who provided incomplete answers, the final sample was composed of 1456 respondents. Table 1 provides a description of the characteristics of the sample, which were distributed according to the age of majority in Canada, either minor (under age 18) or person of full age (age 18 and over). More specifically, the minimum age is 3 years, whereas the maximum age is 61 years. The median is 14 years, the average is approximately 17 years and the standard deviation is 8.66. Note that for minor respondents, the parents were invited to help their child complete the questionnaire.

### 3.2. Impact of the Global Pandemic on the Overall Level of Physical Activity of Athletes and Their Feeling of Well-Being

#### 3.2.1. Overall Level of Physical Activity

Half (50.4%) of respondents found that their overall level of physical activity decreased during the pandemic compared to before. Among these individuals, 17.4% stated that their overall level of physical activity had decreased significantly, whereas 33% stated that it had decreased a little. However, 29.6% of respondents indicated that their level of activity remained the same. Among the remaining 20%, 15.3% of respondents stated that their activity level had increased a little and 4.7% indicated that it had increased significantly.

Independent samples t-tests were conducted to determine if the impact of the pandemic on the overall level of physical activity differed significantly according to certain characteristics. The results showed that there was no significant difference between elite athletes (level AAA or semi-professional) and recreational athletes (level A or AA) (*p* = 0.095), between female and male athletes (*p* = 0.911) and between minors and persons of full age (*p* = 0.658).

#### 3.2.2. Correlation between Physical Activity and Feeling of Well-Being during COVID-19

The average percentage obtained in WHO-5 for all respondents is 65.74%. In total, 17% of respondents have an average below 50%, whereas 83% are above. Independent samples t-tests showed that the percentage obtained in WHO-5 was not significantly different between: (1) female vs. male athletes (*p* = 0.059); (2) elite vs. recreational athletes (*p* = 0.192); and (3) minors vs. persons of full age (*p* = 0.658). For the purpose of verifying if the feeling of well-being differed significantly according to whether an athlete felt that their overall level of physical activity had decreased during the pandemic, remained the same or had increased, a one-way ANOVA was conducted. This ANOVA aiming to evaluate the possible relationship between the level of activity during the global pandemic situation and the total score obtained in items measuring well-being proved to be statistically significant (F(2.1438) = 54.707, *p* < 0.001). In addition, the strength of the relationship is of moderate magnitude according to the η^2^ statistic of 0.071 [13]. In fact, it would appear that the overall level of physical activity would explain approximately 7.1% of the variations in the total well-being score. Table 2 presents the results of the Bonferroni post hoc tests according to a signification level adjusted by the number of comparisons set at *p* < 0.0167 (i.e., *p* < 0.05/3) [14]. A Bonferroni post hoc test was used because the sample sizes of each group were not equal. The results of the comparisons between each group show that the significant differences in the feeling of well-being are between athletes who felt that their overall level of physical activity had decreased ($\bar{x}$ = 61.81/100) and the others. However, there is no significant difference between athletes who felt that their overall level of physical activity had remained the same ($\bar{x}$ = 70.41/100) and those who felt that it had increased ($\bar{x}$ = 68.84/100).

Similarly, a one-way ANOVA evaluating the relationship between the age and the total score obtained in items measuring well-being proved to be statistically significant (F(3.1302) = 24.625, *p* < 0.001; η^2^ = 0.054). The average overall score obtained in WHO-5 among young athletes aged 3 to 12 was the highest (69.36%), whereas that of athletes aged between 18 and 35 years was the lowest (60.20%). Table 3 presents the results of the Bonferroni post hoc tests. The results of the comparisons between each group show that the significant differences in the feeling of well-being are between younger athletes ($\bar{x}$ = 69.36/100) and others. There is also a significant difference between athletes between age 13 and 17 ($\bar{x}$ = 65.74/100) and those between age 18 and 35 ($\bar{x}$ = 60.20/100).

### 3.3. Changes in Athletes Physical Activity Practices in a Global Pandemic Context

Table 4 presents descriptive statistics witnessing certain changes in physical activity practices comparing before and during the global pandemic for all respondents. The last column presents the statistics of athletes who did not practice the activity concerned either before or during the global pandemic, and the other statistics are calculated excluding these respondents. For all activities surveyed, a majority of respondents felt that their level of practice remained the same. Moreover, compared to those who judged that their level of practice decreased, a larger number of respondents deemed having increased their level of practice for all activities surveyed, except swimming. Home exercise was the activity whose level increased by the largest percentage of respondents (50.2%), which was followed by outdoor activities (37.4%) and jogging (37.2%).

Table 5 presents the results of independent samples t-tests conducted in order to determine if the changes in physical activity practices differed significantly according to the respondents’ age and competitive levels. Walking and swimming were not influenced by age or the athletes’ competitive levels. However, persons of full age and elite athletes deemed having significantly increased their level of home exercise and jogging more than minors and recreational athletes. Moreover, persons of full age significantly increased their level of outdoor activities and elite athletes significantly increased their level of wheeled sports. Similarly, other *t*-tests showed that the changes in levels of physical activity did not differ significantly based on the respondents’ sex.

### 3.4. Post-Pandemic Physical Activity Intentions

The last column of Table 6 presents the descriptive statistics for all respondents. The results show that the majority of respondents intend to maintain the same level of post-pandemic practice for all activities surveyed. Moreover, about one-fifth of respondents estimated that their level of practice in these activities will increase when the public health measures implemented to prevent the spread of the virus have been lifted.

Chi-square tests were then conducted to determine if the proportion of respondents who intended to decrease, increase or maintain the same level of post-pandemic practice for the activities surveyed was equal between respondents having reduced and those having increased the level of practice in these activities during the pandemic. The results show that the intentions of the two groups do not differ for jogging and swimming, but they differ for walking, wheeled sports, outdoor activities and home exercise.

A standardized residual post hoc analysis was conducted for each significant chi-square analysis following the procedure recommended by Beasley and Schumacker [15] and by Garcia-Perez and Nunez-Anton [16]. These analyses were used to determine which groups had a different distribution than expected. According to the Bonferroni method, the *p*-value has also been adjusted as a consequence of the number of comparisons made, namely *p* < 0.008 (i.e., *p* < 0.05/6) [14]. The result of the significant chi-square tests whose adjusted residuals were greater than 3.58 (*p* < 0.008; to take into account multiple comparisons) are indicated by an asterisk in Table 6.

The results of these analyses indicate that respondents’ post-pandemic intentions with respect to walking vary according to whether the respondents had decreased or increased their level of walking during the pandemic. In fact, the proportion of respondents who intend to maintain the same level of walking is significantly higher among individuals who increased (56.1% vs. 35.7%) their level of walking during the pandemic, while the proportion of respondents who intend to increase their level of walking is significantly higher among individuals who decreased it (48.7% vs. 31.3%) during the pandemic. At the same time, the intentions of respondents concerning their practice of outdoor activities varies in a similar fashion. More specifically, the proportion of respondents who intend to maintain the same level of practicing outdoor activities is significantly higher among individuals who increased it (54.3% vs. 28.8%) during the pandemic, whereas the proportion of respondents who intend to increase their practice of outdoor activities is significantly higher among individuals who decreased it (61.1% vs. 33.9%) during the pandemic. Moreover, the proportion of respondents who intend to maintain the same level of practice in wheeled sports is significantly higher among individuals who increased (60.3% vs. 38.7%) their level of practice in wheeled sports during the pandemic. However, the proportion of respondents who intend to increase their level of home exercise at home is significantly higher among individuals who decreased it (44.3% vs. 25.9%) during the pandemic.

## 4. Discussion

In the context of COVID-19, the governments imposed unprecedented restrictions, thereby upsetting the normal daily life of many individuals. The measures implemented to limit the transmission of the virus restricted the range of possibilities for practicing physical activity. The impacts of these measures were felt the most among athletes since they made it difficult, if not impossible, to practice their team or individual sports. To maintain the same level of physical activity, they had to find alternatives that complied with the measures implemented. Consequently, in these unusual conditions, different lifestyles were affected, particularly the change in practicing certain physical activities. This article therefore aimed to understand the changes in behavior in terms of athletes’ physical activities in the context of a global pandemic.

The results of this study suggest that the limitations imposed during the pandemic led to half of athletes decreasing their overall level of physical activity. This finding is in line with the results obtained in the study of Castañeda-Babarro et al. [17] which showed that self-reported physical activity decreased significantly during confinement in the Spanish population. This result can be simply explained by the fact that these respondents did not systematically substitute the suspended activities with equivalent activities in order not to see their overall level of activity decrease. In addition, individuals placed in a stressful situation tend to decrease their level of physical activity. In fact, according to the systematic review carried out by Stults-Kolehmainen and Sinha [18], the majority of articles identified showed that the level of stress felt was associated with a reduction in the level of physical activity. This is one of the rare studies that focuses on the influence of stress on the level of physical activity and not on exploring the impact of physical activity on stress reduction. The uncertainty about the pandemic resulted in the prevalence rate of stress proving to be higher in the general population during this period [19]. That said, the increase in the overall level of activity of one-fifth of the respondents could also be a response aiming to combat the stress caused by the pandemic, since, on the contrary, certain individuals exercise to deal with a stressful situation [18]. In addition, in a stressful situation, people who are used to being active, as is the case for our sampling composed of athletes, generally tend to further increase their level of physical activity in response to stress than inactive people [18]. Moreover, this increase can also be linked to the fact that many individuals had additional free time to exercise during the period of confinement [20]. Public health also encouraged citizens to exercise regularly to prevent complications from COVID-19 [20].

The overall average score obtained in WHO-5 for all respondents is 65.74%, while studies of the general population estimated that the average score is generally about 70% [21]. Almost one-fifth of respondents obtained an overall score lower than 50%, whereas this score is often used as a critical threshold to screen individuals at risk for depression [11]. The statistical analyses also showed that the level of psychological well-being was statistically lower among athletes who decreased their level of physical activity during the pandemic. This result goes hand in hand with numerous studies which have shown that physical activity has a positive impact on psychological well-being [22]. The benefits of physical activity prove to be all the more important in a context that contributes to accentuating the symptoms of stress and anxiety felt by many individuals vis-à-vis the disruption in the normal course of their daily life [23]. Nevertheless, in their study, Lesser and Nienhuis [24] found that there were no significant differences in well-being outcomes in the active Canadian population between those who were more active, the same or less active during the pandemic. Given that this study was conducted at the beginning of the pandemic (nationwide restrictions were in place for 50 days), it is possible to hypothesize that the impact of the pandemic on well-being had not yet been felt. Additional studies are needed to better understand the impact of decreased physical activity on well-being during a global pandemic situation. Finally, the statistical analyses showed that athletes aged between 18 and 35 obtained the lowest average level of well-being ($\bar{x}$ = 60.20/100). This result is consistent with the finding obtained in the study of Rochelle et al. [25], which show that among Australians, the younger age cohort (18–29 years) reported significantly poorer general well-being during the pandemic than the older age groups. In addition, the level of psychological well-being was significantly higher among young athletes between 3 and 12 years of age than for all other age groups. These results go hand in hand with the review of literature containing studies on the relationship between age and well-being conducted by López et al. [26], which supports a U-shaped relationship where young people and seniors are generally happier than middle-aged people. This result is also consistent with the data gathered during the pandemic by the National Center for Health Statistics which showed that young adults aged 18 to 29 were more likely to suffer from anxiety than other adult age groups [27,28].

Considering that half of the respondents deemed that their overall level of activity remained the same or increased, these individuals were forced to find alternatives in order to be able to maintain the same volume of physical activity. Among all the activities surveyed, only swimming obtained a higher percentage of respondents who deemed having decreased their participation in this activity. Even though swimming pools were closed for a shorter period than many other indoor public places, such as gyms, it is still the most regulated activity among the six activities surveyed (see Appendix A). Since they could no longer swim in indoor pools, athletes who practiced swimming before the pandemic were restricted to reducing, and even completely ending their level of participation in winter. The present study also showed that the changes in the level of physical activity differed significantly based on the respondents’ competitive levels. Elite athletes significantly increased their level of exercise at home, their practice of wheeled sports and jogging more than recreational athletes. These results can be explained by the fact that among the majority of elite athletes, the volume, intensity and frequency of pre-pandemic training was higher than among recreational athletes. Since they could no longer follow their usually charged training programs, elite athletes tried to find alternatives in order to reduce the weight of the pandemic on their performance. Among other things, many sporting clubs offered home training programs for their elite athletes and organized videoconferences for online training sessions led by their coaches in order to reduce the risk of athletes being exposed to a level of detraining, i.e., the partial or complete loss of morphological and psychological adaptations induced by training, due to insufficient or inappropriate training stimuli [29].

### 4.1. Changes in Physical Activity Practices Resulting from the Pandemic: Temporary or Sustainable?

This study shed light on numerous changes in physical activity practices resulting from the pandemic. Most of these changes do not result from deliberate individual decisions but are dependent on exceptional circumstances linked to the pandemic. It is therefore legitimate to wonder if the pandemic will have a sustainable and positive impact on athletes’ behaviors or if these behaviors will be replaced by pre-crisis habits. The results of this study have shown that for all activities surveyed, the majority of respondents who deem having increased their level of activity believe that these changes will last, i.e., that they will maintain this same level of post-pandemic activity. Certain respondents even consider that the increase in their level of practice will continue to grow. Therefore, it is more than just a temporary substitution. Having more free time, it is possible that these athletes discover new passions [30] or they felt the benefits of these activities during the confinement.

That said, knowing that there could be a difference between the behavioral intentions declared and the behaviors observed [31], the question can also be asked if these intentions will actually materialize. In this context, opinions diverge as to the sustainability of the new behaviors adopted during the crisis: some are in favor of a sustainable change, while others presume a return to pre-pandemic life. [32]. In order for athletes who increased their level of participation in certain activities during the pandemic to maintain this habit after the pandemic, care must be taken to ensure that the perceived benefits associated with it do not diminish with time [32]. For example, certain athletes will have had the opportunity to observe, repeatedly, the numerous benefits of outdoor activity [33] allowing them, according to a connectionist perspective, to learn associations between behaviors and rewards in a given environment [32,34]. Nevertheless, given that the changes in behaviors that appeared out of necessity during the pandemic are intimately related to the context of the crisis, after-COVID could provoke a return to pre-crisis behaviors, since, still according to a connectionist perspective, the habits are strongly dependent on the context [32]. The pandemic represents a major change in context, which might suggest that the return to a pre-crisis context will lead to pre-crisis behaviors.

This same reasoning goes hand-in-hand with the fact that most athletes who reduced their level of practice in certain activities during the pandemic consider that their level of practice will start to increase again after the pandemic and that they will therefore resume their pre-pandemic habits. The change of context could have a negative impact on habits for certain physical activities whether it is associated with the reduction in movement, loss of motivation, repercussions of school closings on parents’ free time, etc. Therefore, the return to normal in a stable environment could lead to a return to old habits [35]. That said, to succeed in returning to a higher level of physical activity, athletes will have to fight a certain inertia that this forced period of immobilization will have somewhat ingrained [36].

### 4.2. Limits and Future Research Avenues

This study contains three main limitations. First, resorting to self-reported measures was critical for the evaluation of physical activity, since individuals tend to overestimate their level of physical activity [37]. Second, the absence of data on well-being before the pandemic limits the capacity for this study to witness changes in the level of well-being attributed to the pandemic. Third, certain questions aiming to compare retrospectively and subjectively the level of physical activity before and during the pandemic could have caused a recall bias.

Given these limits and conditions in which this study was carried out, it would be interesting to further explore certain research avenues. Future research should use more objective measures of physical activity to determine the impact of a pandemic on the level of athletes’ physical activity. These measures could be regularly collected in order to facilitate pre- and post-pandemic comparisons. Similarly, it would be interesting to study the longitudinal impact of sanitary measures on athletes’ level of detraining, performances and motivations. Finally, complementary research could explore the effects of resuming sports activities on athletes’ psychological well-being.

## 5. Conclusions

In conclusion, the results of this study show that the pandemic will have had the impact of creating an abrupt forced shutdown of numerous activities, leading to a reduction in the overall level of physical activity of the athletes questioned, causing a deterioration in their psychological well-being. In anticipation of a new wave of infections or even a new global pandemic, it is important to implement strategies that favor practicing physical activities in a pandemic context. For example, this could come in the form of reorganizing current or future public spaces to favor walking, jogging and wheeled sports, because of the rise in popularity of these activities during the pandemic. Moreover, individuals should be encouraged to practice outdoor activities in accordance with sanitary measures ideally by keeping parks and green spaces open and accessible to the population, even in a period of confinement. Finally, the results of this study show that almost all individuals who increased their practice of wheeled sports during the pandemic believe that it will continue after the pandemic. Therefore, this is a great opportunity to promote active transportation among athletes by commending its many benefits on physical and mental well-being and on the environment in order to ensure that the perception of the benefits associated with it does not decrease with time. Studies on crises situations have shown that great adversity imposed on societies can be pivotal moments to improve them [32]. Changes in behaviors arose during the pandemic, some of which favor a more active lifestyle and which would be preferable if they continued.

## Figures and Tables

**Table 1:** Sports profile of participants (*n* = 1456).

	Minors No. (%) 950 (72.2)	Persons of Full Age No. (%) 366 (27.8)	Total No. (%) 1456 (100)
Sex			
Female	337 (35.5)	187 (51.1)	546 (40.1)
Male	612 (64.5)	179 (48.9)	815 (59.9)
Competitive level			
A (local division 1-2-3)	557 (59.6)	251 (69.5)	832 (62.0)
AA	246 (23.3)	83 (23.0)	340 (25.4)
AAA	130 (13.9)	22 (6.1)	162 (12.1)
Semi-pro	2 (0.2)	5 (1.4)	7 (0.5)

Note: “No.” = number.

**Table 2:** Bonferroni multiple comparison test performed on three levels of overall physical activity in relation to the feeling of well-being (*n* = 1441).

Activity Level	Average	Standard Deviation	Reduced	Remained the Same	Increased
Reduced	61.81	15.2	NA	* (*p* < 0.001)	* (*p* < 0.001)
Remained the same	70.41	13.525		NA	NS
Increased	68.84	14.396			NA

Note: “NA”= not applicable. “*” = significant according to adjusted *p*-value, *p* < 0.0167. “NS” = non-significant.

**Table 3:** Bonferroni multiple comparison test performed on the four age categories in relation to the feeling of well-being (*n* = 1306).

Age	Average	Standard Deviation	Age 3–12	Age 13–17	Age 18–35	Age 36–61
3–12	69.36	13.595	NA	* (*p* = 0.001)	* (*p* < 0.001)	* (*p* = 0.003)
13–17	65.74	14.592		NA	* (*p* < 0.001)	NS
18–35	60.20	16.322			NA	NS
36–61	62.59	13.846				NA

Note: “*” = significant according to adjusted *p*-value, *p* < 0.0125.

**Table 4:** Comparisons between physical activity practices before and during the global pandemic.

	Much Less than Before No. (%)	A Little Less than Before No. (%)	As Much as Before No. (%)	A Little More than Before No. (%)	Much More than Before No. (%)	Do Not Practice This Activity No. (%)
Walking	68 (5.2)	98 (7.6)	787 (60.7)	233 (18.0)	110 (8.5)	160 (11.0)
Wheeled sports	42 (3.3)	81 (6.3)	748 (58.6)	253 (19.8)	153 (12.0)	179 (12.3)
Outdoor activities	57 (4.2)	152 (11.2)	640 (47.2)	330 (24.4)	176 (13.0)	98 (6.7)
Home exercise	25 (2.1)	70 (5.8)	505 (41.9)	363 (30.1)	242 (20.1)	248 (17.1)
Jogging	38 (3.4)	81 (7.2)	586 (52.2)	284 (25.3)	133 (11.9)	332 (22.8)
Swimming	124 (14.5)	105 (12.3)	518 (60.7)	68 (8.0)	38 (4.5)	601 (41.3)

**Table 5:** Comparisons between physical activity practices before and during the global pandemic according to age and competitive level.

	Minor M ± SD	Person of Full Age M ± SD	*p*-Value	Recreational M ± SD	Elite M ± SD	*p*-Value
Walking	3.15	3.21	NS (0.33)	3.16	3.27	NS (0.171)
Wheeled sports	3.34	3.24	NS (0.138)	3.30	3.48	0.03
Outdoor activities	3.26	3.46	0.001	3.31	3.35	NS (0.651)
Home exercise	3.55	3.73	0.006	3.54	3.98	<0.001
Jogging	3.28	3.50	<0.001	3.29	3.65	<0.001
Swimming	2.73	2.79	NS (0.495)	2.77	2.66	NS (0.275)

Note: Scores based on means of scale ranging from 1 (Not at all) to 5 (Extremely).

**Table 6:** Results of post-pandemic physical activity intentions.

	Reduced during the Pandemic No. (%)	Increased during the Pandemic No. (%)	X^2^	Total No. (%)
Walking				
Will decrease	24 (15.6)	43 (12.6)	<0.001	86 (6.6)
Will remain the same *	55 (35.7)	192 (56.1)		961 (74)
Will increase *	75 (48.7)	107 (31.3)		251 (19.3)
Wheeled sports				
Will decrease	19 (16.0)	34 (8.5)	<0.001	68 (5.3)
Will remain the same *	46 (38.7)	242 (60.3)		975 (75.8)
Will increase	54 (45.4)	125 (31.2)		244 (19.0)
Outdoor activities				
Will decrease	20 (10.1)	59 (11.8)	<0.001	88 (6.5)
Will remain the same *	57 (28.8)	272 (54.3)		878 (65.0)
Will increase *	121 (61.1)	170 (33.9)		384 (28.4)
Home exercise				
Will decrease	18 (20.5)	197 (32.9)	0.001	238 (19.8)
Will remain the same	31 (35.2)	246 (41.1)		718 (59.7)
Will increase *	39 (44.3)	155 (25.9)		247 (20.5)
Jogging				
Will decrease	28 (24.3)	87 (21.2)	NS	138 (12.2)
Will remain the same	40 (34.8)	178 (43.4)		728 (64.5)
Will increase	47 (40.9)	145 (35.4)		262 (23.2)
Swimming				
Will decrease	46 (21.3)	12 (11.4)	NS	70 (8.2)
Will remain the same	65 (30.1)	48 (45.7)		596 (69.8)
Will increase	105 (8.6)	45 (42.9)		188 (2.0)

Note: The X^2^ values represent chi-square tests of independence. “*” = These categories differ from the expected frequency, according to adjusted *p*-value, *p* < 0.008.

## Data Availability

The data presented in this study are available from the corresponding author on reasonable request.

## Appendix A

**Table A1:** Physical activity-related measures adopted in Quebec to control the COVID-19 pandemic.

Dates	Measures Adopted to Control COVID-19 Pandemic	Restrictive	Ease
2020			
14 March	People 70 years and older are asked to stay home.	√	
15 March	Closing of several public places including training centers, swimming pools, arenas and ski centers.	√	
15 March	People in Quebec are being asked to limit their travel.	√	
16 March	Daycare and school closures until 27 March.	√	
21 March	No indoor or outdoor gatherings are allowed.	√	
22 March	Extension of school and daycare closures until 1 May.	√	
24 March	Closure of all non-essential until 13 April.	√	
5 April	All non-essential businesses must remain closed until 4 May.	√	
10 April	Cancellation of all sporting and cultural events until 31 August.	√	
11 May	Reopening of preschools, elementary schools and daycare services, except those in the MMC.		√
20 May	Non-contact individual recreational sports (e.g., golf, canoeing, hiking) allowed in all regions.		√
20 May	Progressive access to some SEPAQ territories.		√
22 May	Authorization of outdoor gatherings under certain conditions (max. 10 people from a maximum of 3 households).		√
30 May	Opening of outdoor public spaces, such as outdoor pools and park play modules.		√
8 June	Resumption of team sports training (soccer, baseball, hockey…).		√
15 June	Resumption of professional sports and horseracing activities.		√
22 June	Opening of summer camps.		√
22 June	Indoor and outdoor gatherings in specific public places are permitted for up to 50 people.		√
22 June	Resumption of team sports games.		√
22 June	Reopening of indoor sports facilities as well as public and private beaches.		√
3 July	Resumption of all sectors of economic activity with the exception of: festivals and major events; regular summer camps with overnight stays; fighting in a sporting context.		√
3 August	The maximum number of people who can attend indoor or outdoor events in public venues increased from 50 to 250.		√
2 September	Resumption of combat sports (e.g., karate, taekwondo, boxing, judo), without competition.		√
14 September	Resumption of extracurricular activities, Sports studies and Arts studies.		√
1 October	Restriction of all contacts for a period of 28 days.	√	
8 October	Organized sports and recreational activities not permitted; closure of gyms and fitness centers.	√	
16 October	Reopening of the alpine ski centers.		√
6 October	Winter activities offered at authorized snowmobile and outdoor centers; access to relays and shelters of the large snowmobile trail network.		√
17 December	Group sports, cultural and recreational activities permitted outdoors in public places (including classes, training and guided activities), alone, in pairs, in families or in groups of up to 8 people and a supervisor, provided that people maintain a distance of 2 m between them.		√
25 December	Non-essential businesses will be required to close from 25 December 2020 to 10 January 2021.	√	
2021			
7 January	Extension of the closure of non-essential businesses until 8 February.	√	
8 February	In the orange zone, reopening of gyms for individual training and indoor sports and leisure activities (individual practice, in duo or with occupants of the same private residence).		√
26 February	Reopening of the pools and arenas for free practice and individual or duo training with an instructor. Eight people from different residences can practice sports and recreational activities outside in public places (plus one person for supervision or animation).		√
15 March	Face-to-face extracurricular activities and field trips may resume in preschool, elementary and secondary schools.		√
26 March	Allowing non-contact indoor activities in any facility, including all sports arenas or training rooms, with limited seating capacity. Reopening of indoor pools in hotels.		√
1 April	Closure of primary and secondary schools (e-learning) and non-essential businesses.	√	
1 April	Outdoor sports or leisure activities permitted only with persons residing at the same address or by a group of eight persons keeping physical distancing.	√	
8 April	Indoor public places for recreation and sports, except swimming pools, ice skating rinks and places for playing tennis and badminton. Closed gyms.	√	
14 April	When engaging in outdoor and indoor recreational activities or team sports, face masks are required.	√	
11 June	Supervised outdoor sports and recreation permitted in groups of 25. Red and orange zones: non-contact sports. Yellow zone: short contact sports.		√
17 June	Auditoriums and indoor stadiums with assigned seating are allowed to present shows and sporting events before an audience of up to 3500 people.		√
12 July	Distance lowered from 2 to 1 m, both indoors and outdoors, except for singing activities and high intensity exercise in gyms.	√	
24 August	Obligation to present the vaccination passport for certain extracurricular physical and athletic activities for high school students.	√	
8 October	Relaxation of sanitary measures for concert halls, sports and cultural auditoriums and organized public gatherings.		√

Inspired by the COVID-19 timeline of The Institut national de santé publique du Québec (INSPQ) [4].

## References

[1] WHO Chronologie De L’action De L’oms Face à La COVID-19. https://www.who.int/fr/news/item/29-06-2020-covidtimeline.

[2] Bordeleau J.-L. Le Canada Parmi Les Pays Les Plus Sévères Quant Aux Règles Pour La COVID-19. https://www.ledevoir.com/societe/sante/671157/le-canada-parmi-les-pays-les-plus-severes-quant-aux-regles-pour-la-covid-19.

[3] Dekker J., Macdonald R. COVID-19 Restrictions Index Update. https://www150.statcan.gc.ca/n1/pub/36-28-0001/2022008/article/00002-eng.htm.

[4] INSPQ Ligne Du Temps COVID-19 Au Québec. https://www.inspq.qc.ca/covid-19/donnees/ligne-du-temps.

[5] WHO Activité Physique. https://www.who.int/fr/news-room/fact-sheets/detail/physical-activity#:~:text=Les%20recommandations%20mondiales%20pr%C3%A9conisent%20au,d’intensit%C3%A9%20soutenue%20par%20semaine.

[6] Chekroud S.R., Gueorguieva R., Zheutlin A.B., Paulus M., Krumholz H.M., Krystal J.H., Chekroud A.M. (2018). Association between physical exercise and mental health in 1·2 million individuals in the USA between 2011 and 2015: A cross-sectional study. Lancet Psychiatry.

[7] Institut de la statistique du Québec Québec Survey on Physical Activity and Sports, 2018–2019. https://statistique.quebec.ca/fr/enquetes/realisees/enquete-quebecoise-sur-lactivite-physique-et-le-sport-eqaps-2018-2019.

[8] Taylor H.L., Jacobs D.R., Schucker B., Knudsen J., Leon A.S., Debacker G. (1978). A questionnaire for the assessment of leisure time physical activities. J. Chronic Dis..

[9] Montoye H.J. (1996). Measuring physical activity and energy expenditure. Hum. Kinet..

[10] Nolin B., Prud’homme D., Godin G., Hamel D. (2002). Enquête québécoise sur l’activité physique et la santé. https://bel.uqtr.ca/id/eprint/551/1/6-19-1668-20070109-1.pdf.

[11] Topp C.W., Otergaard S.D., Sondergaard S., Bech P. (2015). The WHO-5 Well-Being Index: A systematic review of the literature. Psychother. Psychosom..

[12] George D., Mallery P. (2019). IBM SPSS Statistics 26 Step by Step: A Simple Guide and Reference.

[13] Cohen J. (2013). Statistical Power Analysis for the Behavioral Sciences.

[14] Bland J.M., Altman D.G. (1995). Multiple significance tests: The Bonferroni method. BMJ.

[15] Beasley T.M., Schumacker R.E. (1995). Multiple regression approach to analyzing contingency tables: Post hoc and planned comparison procedures. J. Exp. Educ..

[16] Garcia-Perez M.A., Nunez-Anton V. (2003). Cellwise residual analysis in two-way contingency tables. Educ. Psychol. Meas..

[17] Castañeda-Babarro A., Arbillaga-Etxarri A., Gutiérrez-Santamaría B., Coca A. (2020). Physical activity change during COVID-19 confinement. Int. J. Environ. Res. Public Health.

[18] Stults-Kolehmainen M.A., Sinha R. (2014). The effects of stress on physical activity and exercise. Sport. Med..

[19] Lakhan R., Agrawal A., Sharma M. (2020). Prevalence of depression, anxiety, and stress during COVID-19 pandemic. J. Neurosci. Rural Pract..

[20] Lim M.A. (2021). Exercise addiction and COVID-19-associated restrictions. J. Ment. Health.

[21] INSPQ Fiches Pour Les Instruments De Mesure Standardisés Recommandés. https://www.inspq.qc.ca/boite-outils-pour-la-surveillance-post-sinistre-des-impacts-sur-la-sante-mentale/instruments-de-mesure-standardises/fiches-pour-les-instruments-de-mesure-standardises-recommandes/bien-etre.

[22] McAuley E., Rudolph D. (1995). Physical activity, aging, and psychological well-being. J. Aging Phys. Act..

[23] Maugeri G., Castrogiovanni P., Battaglia G., Pippi R., D’Agata V., Palma A., Di Rosa M., Musumeci G. (2020). The impact of physical activity on psychological health during COVID-19 pandemic in Italy. Heliyon.

[24] Lesser I.A., Nienhuis C.P. (2020). The impact of COVID-19 on physical activity behavior and well-being of Canadians. Int. J. Environ. Res. Public Health.

[25] Eime R., Harvey J., Charity M., Elliott S., Drummond M., Pankowiak A., Westerbeek H. (2022). The impact of COVID-19 restrictions on perceived health and wellbeing of adult Australian sport and physical activity participants. BMC Public Health.

[26] López Ulloa B.F., Møller V., Sousa-Poza A. (2013). How does subjective well-being evolve with age? A literature review. J. Popul. Ageing.

[27] Knepple Carney A., Graf A.S., Hudson G., Wilson E. (2021). Age moderates perceived COVID-19 disruption on well-being. Gerontologist.

[28] Center for Disease Control and Prevention Mental health: Household Pulse Survey. https://www.cdc.gov/nchs/covid19/pulse/mental-health.htm.

[29] Sarto F., Impellizzeri F.M., Spörri J., Porcelli S., Olmo J., Requena B., Suarez-Arrones L., Arundale A., Bilsborough J., Buchheit M. (2020). Impact of potential physiological changes due to COVID-19 home confinement on athlete health protection in elite sports: A call for awareness in sports programming. Sport. Med..

[30] Dvorsky M.R., Breaux R., Becker S.P. (2021). Finding ordinary magic in extraordinary times: Child and adolescent resilience during the COVID-19 pandemic. Eur. Child Adolesc. Psychiatry.

[31] Sheeran P., Webb T.L. (2016). The intention–behavior gap. Soc. Personal. Psychol. Compass.

[32] Trespeuch L., Robinot É., Botti L., Bousquet J., Corne A., De Ferran F., Durif F., Ertz M., Fontan J.-M., Giannelloni J.-L. (2021). Allons-nous vers une société plus responsable grâce à la pandémie de COVID-19?. Nat. Sci. Soc..

[33] Jackson S.B., Stevenson K.T., Larson L.R., Peterson M.N., Seekamp E. (2021). Outdoor activity participation improves adolescents’ mental health and well-being during the COVID-19 pandemic. Int. J. Environ. Res. Public Health.

[34] Wood W., Tam L., Witt M.G. (2005). Changing circumstances, disrupting habits. J. Personal. Soc. Psychol..

[35] Maltagliati S., Rebar A., Fessler L., Forestier C., Sarrazin P., Chalabaev A., Sander D., Sivaramakrishnan H., Orsholits D., Boisgontier M.P. (2021). Evolution of physical activity habits after a context change: The case of COVID-19 lockdown. Br. J. Health Psychol..

[36] Gouin J.-P. Pour Un Québec Actif, Inclusif Et Énergique. https://www.acfas.ca/publications/magazine/2020/04/quebec-actif-inclusif-energique-covid-19.

[37] Dyrstad S.M., Hansen B.H., Holme I.M., Anderssen S.A. (2014). Comparison of self-reported versus accelerometer-measured physical activity. Med. Sci. Sport. Exerc..

